# (2*E*,7*E*)-2,7-Bis[(thio­phen-2-yl)methyl­idene]cyclo­hepta­none

**DOI:** 10.1107/S1600536814011866

**Published:** 2014-05-31

**Authors:** C. Nithya, M. Sithambaresan, S. Prathapan, M. R. Prathapachandra Kurup

**Affiliations:** a Department of Applied Chemistry, Cochin University of Science and Technology, Kochi 682 022, India; b Department of Chemistry, Faculty of Science, Eastern University, Sri Lanka, Chenkalady, Sri Lanka

## Abstract

The whole molecule of the title compound, C_17_H_16_OS_2_, is generated by two-fold rotational symmetry. The carbonyl C and O atoms of the cycloheptanone ring lie on the twofold rotation axis which bisects the opposite –CH_2_–CH_2_– bond of the ring. The mol­ecule exists in an *E*,*E* conformation with respect to the C=C double bond. The cyclo­hepta­none ring exhibits a twisted chair conformation and its mean plane makes a dihedral angle of 50.12 (19)° with the planes of the thio­phene rings. The two S atoms are in an *anti* arrangement with respect the carbonyl O atom and the dihedral angle between the two thio­phene ring planes is 69.38 (7)°. In the molecule, there are two intramolecular C—H⋯S hydrogen bond, forming *S*(6) ring motifs. In the crystal, inversion dimers are generated *via* pairs of C—H⋯O hydrogen bonds. These dimers are inter­connected by another inter­action of the same kind with a neighbouring mol­ecule, forming a mol­ecular chain along the *c*-axis direction.

## Related literature   

For applications of thio­phene derivatives in conducting polymers and biology, see: Kolodziejczyk *et al.* (2013 ▶); Mishra *et al.* (2011 ▶). For the synthesis of related compounds, see: Alkskas *et al.* (2013 ▶). For related structures, see: Liang *et al.* (2007 ▶); Layana *et al.* (2014 ▶).
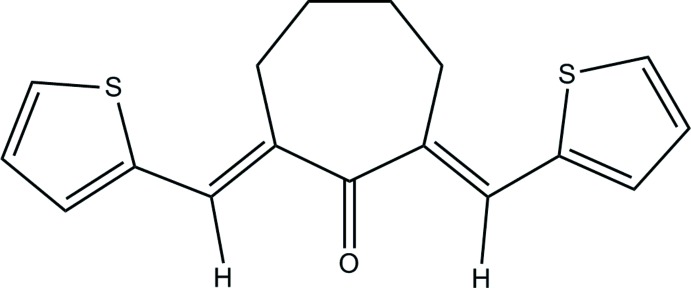


## Experimental   

### 

#### Crystal data  

C_17_H_16_OS_2_
*M* *_r_* = 300.44
Orthorhombic, 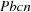

*a* = 16.383 (2) Å
*b* = 11.6119 (14) Å
*c* = 7.8213 (7) Å
*V* = 1487.9 (3) Å^3^
*Z* = 4
Mo *K*α radiation
μ = 0.35 mm^−1^
*T* = 296 K
0.40 × 0.25 × 0.20 mm

#### Data collection  

Bruker APEXII CCD diffractometer
Absorption correction: multi-scan (*SADABS*; Bruker, 2004 ▶) *T*_min_ = 0.873, *T*_max_ = 0.933
4249 measured reflections
1296 independent reflections
970 reflections with *I* > 2σ(*I*)
*R*_int_ = 0.025

#### Refinement  

*R*[*F* ^2^ > 2σ(*F* ^2^)] = 0.049
*wR*(*F* ^2^) = 0.162
*S* = 1.03
1296 reflections
92 parameters
H-atom parameters constrained
Δρ_max_ = 0.20 e Å^−3^
Δρ_min_ = −0.33 e Å^−3^

### 

Data collection: *APEX2* (Bruker, 2004 ▶); cell refinement: *APEX2* and *SAINT* (Bruker, 2004 ▶); data reduction: *SAINT* and *XPREP* (Bruker, 2004 ▶); program(s) used to solve structure: *SHELXS97* (Sheldrick, 2008 ▶); program(s) used to refine structure: *SHELXL97* (Sheldrick, 2008 ▶); molecular graphics: *ORTEP-3 for Windows* (Farrugia, 2012 ▶) and *DIAMOND* (Brandenburg, 2010 ▶); software used to prepare material for publication: *SHELXL97* and *publCIF* (Westrip, 2010 ▶).

## Supplementary Material

Crystal structure: contains datablock(s) I, global. DOI: 10.1107/S1600536814011866/zl2588sup1.cif

Structure factors: contains datablock(s) I. DOI: 10.1107/S1600536814011866/zl2588Isup2.hkl

Supporting information file. DOI: 10.1107/S1600536814011866/zl2588Isup3.cml

CCDC reference: 1004531

Additional supporting information:  crystallographic information; 3D view; checkCIF report

## Figures and Tables

**Table 1:** Hydrogen-bond geometry (Å, °)

*D*—H⋯*A*	*D*—H	H⋯*A*	*D*⋯*A*	*D*—H⋯*A*
C7—H7*A*⋯S1	0.97	2.53	3.205 (3)	126
C7—H7*B*⋯O1^i^	0.97	2.53	3.282 (3)	135

Symmetry code: (i) 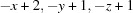.

**Fig. 1.**
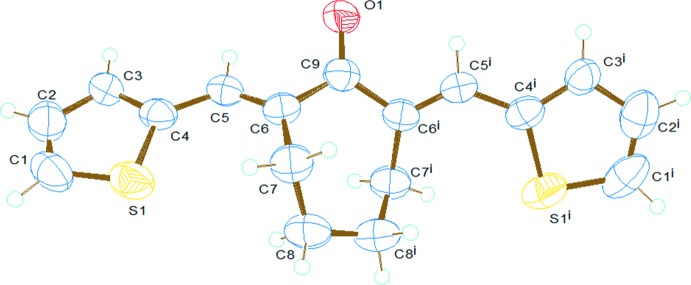
ORTEP view of the compound, drawn with 50% probability displacement ellipsoids for the non-H atoms. Symmetry operator (i): (i) -x+2, y, -z+1/2.

**Fig. 2.**
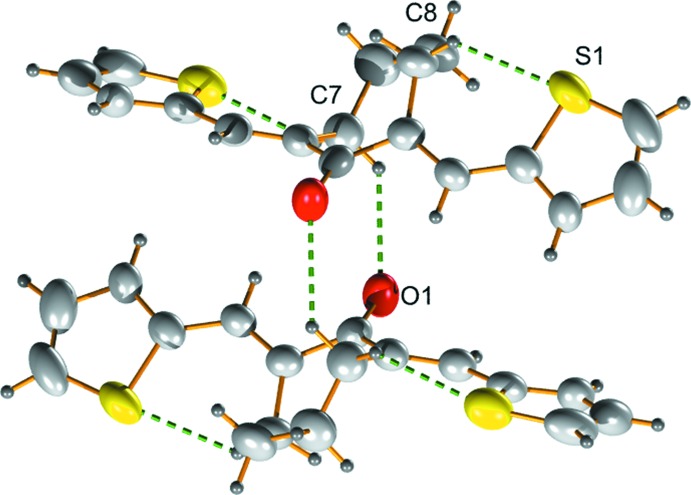
Centrosymmetric dimer formed by means of C–H···O interaction found in the title compound C17H16OS2.

**Fig. 3.**
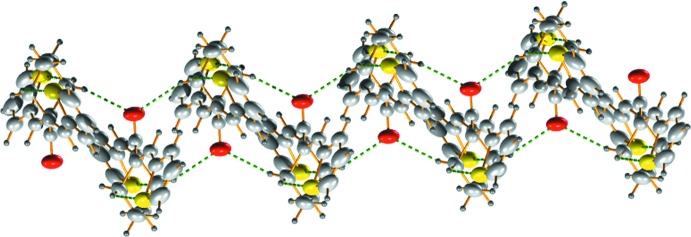
C—H···O interactions forming a 1-D molecular chain along the c axis.

**Fig. 4.**
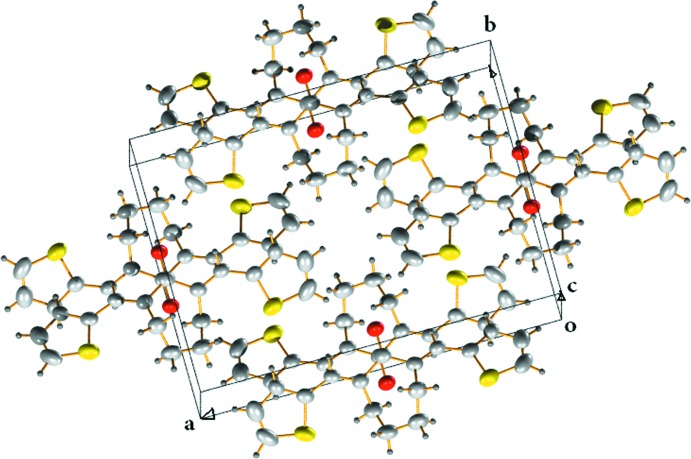
A view of the unit cell along the c axis.

## supplementary crystallographic information

### 1. Comment

Thiophenes are often a subject of considerable interest due to their numerous and interesting conducting properties as polymers and for their biological properties. The incorporation of rigid spacers into the thiophene backbone is anticipated to offer several distinct advantages. A polymer can be extended to afford a more planar conformation through diminished steric effects so that a maximum degree of delocalization of the π electrons is achieved (Kolodziejczyk *et al.*, 2013). Some thiophene derivatives were also evaluated for anti-cancer activity against PC-3 cell lines, for *in vitro* antioxidant potential and for β-glucuronidase and α-glucosidase inhibitory activities. Some also showed a potent DPPH radical scavenging antioxidant activity (Mishra *et al.*, 2011).

The title compound (Scheme 1, Fig. 1) crystallizes in the orthorhombic space group *Pbcn*. It has an *E* configuration with respect to the C6=C7 bond on both sides of the cycloheptanone ring. The central moiety (cycloheptanone) exists in a twisted chair form making a dihedral angle of 50.12 (19)° with the thiophene ring. The C9–O1 (1.227 (5) Å) bond distance is very close to the reported bond lengths (1.222 (3) Å) of a keto group of a similar structure (Liang *et al.*, 2007). The two sulfur atoms are in an *anti* arrangement with respect to the carbonyl O atom and the dihedral angle between the two five-membered thiophene ring planes is 69.38 (7)°. There are no classical hydrogen bond interactions present in the structure. However, an intramolecular H bond interaction between one of the H atoms at C7 atom and the S1 atom forms a five membered ring with a D···A distance of 3.205 (3) Å (Fig. 2) within the molecule whereas two C—H···O intermolecular hydrogen bond interactions with a D···A distance of 3.282 (3) Å (Table 1) between H7A and O1 generate a centrosymmetric dimer (Layana *et al.*, 2014) and also chain these centrosymmetric dimers into a 1-D chain along the *c* axis in the lattice (Fig. 3). In addition to this, there are two very weak π···π interactions found in the crystal between the thiophene rings with centroid-centroid distances of greater than 4 Å. Fig. 4 shows the packing of the title compound along *c* axis.

### 2. Experimental

The title compound was prepared by adapting a reported procedure (Alkskas *et al.*, 2013). To a mixture of cycloheptanone (0.50 g, 4.4 mmol) and thiophene-2-carboxaldehyde (1.01 g, 8.7 mmol) in methanol (25 ml) taken up in a 100 ml flask, potassium hydroxide pellets (0.5 g, 8.7 mmol) were added and the reaction mixture was stirred at room temperature for 15 minutes whilst a yellow product separated out. The mixture was heated in a hot water bath at 60 °C for 6 h. until an appreciable amount of solid formed. The flask was then cooled in ice and the precipitate that separated out was collected by vacuum filtration. The crude product was washed several times with ice cold 1 ml portions of ethanol. Recrystallization from methanol gave diffraction quality crystals (yield 98%). m.p: 144–146 °C.

IR (KBr, ν in cm^-1^): 2906, 1598, 1401,1297. ^1^H NMR (400 MHz, CDCl_3_, δ, p.p.m): 7.58 (d, 1H), 7.46(d, 1H), 7.31(t, 1*H*), 7.10 (s, 1*H*), 2.80 (t, 2*H*), 1.95 (m, 2H).

### 3. Refinement

All H atoms on C were placed in calculated positions, guided by difference maps, with C—H bond distances of 0.93–0.97 Å. H atoms were assigned *U*_iso_(H) values of 1.2U_eq_(carrier).

### Crystal data

**Table:**

C_17_H_16_OS_2_	*F*(000) = 632
*M**_r_* = 300.44	*D*_x_ = 1.341 Mg m^−^^3^
Orthorhombic, *P**b**c**n*	Mo *K*α radiation, λ = 0.71073 Å
Hall symbol: -P 2n 2ab	Cell parameters from 1331 reflections
*a* = 16.383 (2) Å	θ = 2.5–28.4°
*b* = 11.6119 (14) Å	µ = 0.35 mm^−^^1^
*c* = 7.8213 (7) Å	*T* = 296 K
*V* = 1487.9 (3) Å^3^	Block, colorless
*Z* = 4	0.40 × 0.25 × 0.20 mm

### Data collection

**Table:**

Bruker APEXII CCD diffractometer	1296 independent reflections
Radiation source: fine-focus sealed tube	970 reflections with *I* > 2σ(*I*)
Graphite monochromator	*R*_int_ = 0.025
Detector resolution: 8.33 pixels mm^-1^	θ_max_ = 25.0°, θ_min_ = 2.5°
ω and φ scan	*h* = −19→9
Absorption correction: multi-scan (*SADABS*; Bruker, 2004)	*k* = −12→13
*T*_min_ = 0.873, *T*_max_ = 0.933	*l* = −9→5
4249 measured reflections	

### Refinement

**Table:**

Refinement on *F*^2^	Primary atom site location: structure-invariant direct methods
Least-squares matrix: full	Secondary atom site location: difference Fourier map
*R*[*F*^2^ > 2σ(*F*^2^)] = 0.049	Hydrogen site location: inferred from neighbouring sites
*wR*(*F*^2^) = 0.162	H-atom parameters constrained
*S* = 1.03	*w* = 1/[σ^2^(*F*_o_^2^) + (0.0941*P*)^2^ + 0.5613*P*] where *P* = (*F*_o_^2^ + 2*F*_c_^2^)/3
1296 reflections	(Δ/σ)_max_ < 0.001
92 parameters	Δρ_max_ = 0.20 e Å^−^^3^
0 restraints	Δρ_min_ = −0.33 e Å^−^^3^

### Special details

**Table:**

Geometry. All e.s.d.'s (except the e.s.d. in the dihedral angle between two l.s. planes) are estimated using the full covariance matrix. The cell e.s.d.'s are taken into account individually in the estimation of e.s.d.'s in distances, angles and torsion angles; correlations between e.s.d.'s in cell parameters are only used when they are defined by crystal symmetry. An approximate (isotropic) treatment of cell e.s.d.'s is used for estimating e.s.d.'s involving l.s. planes.
Refinement. Refinement of *F*^2^ against ALL reflections. The weighted *R*-factor *wR* and goodness of fit *S* are based on *F*^2^, conventional *R*-factors *R* are based on *F*, with *F* set to zero for negative *F*^2^. The threshold expression of *F*^2^ > σ(*F*^2^) is used only for calculating *R*-factors(gt) *etc*. and is not relevant to the choice of reflections for refinement. *R*-factors based on *F*^2^ are statistically about twice as large as those based on *F*, and *R*- factors based on ALL data will be even larger.

### Fractional atomic coordinates and isotropic or equivalent isotropic displacement parameters (Å^2^)

**Table:**

	*x*	*y*	*z*	*U*_iso_*/*U*_eq_	
C1	0.6448 (2)	0.2975 (4)	0.3099 (5)	0.0826 (12)	
H1	0.6039	0.2459	0.3403	0.099*	
C2	0.6311 (2)	0.3968 (4)	0.2293 (5)	0.0817 (11)	
H2	0.5793	0.4217	0.1976	0.098*	
C3	0.70218 (17)	0.4600 (3)	0.1968 (4)	0.0570 (8)	
H3	0.7028	0.5309	0.1413	0.068*	
C4	0.77149 (18)	0.4051 (2)	0.2567 (3)	0.0541 (7)	
C5	0.85397 (16)	0.4491 (2)	0.2406 (3)	0.0503 (7)	
H5	0.8575	0.5190	0.1831	0.060*	
C6	0.92557 (17)	0.4079 (2)	0.2929 (3)	0.0502 (7)	
C7	0.9388 (2)	0.2988 (3)	0.3929 (4)	0.0635 (8)	
H7A	0.8902	0.2839	0.4604	0.076*	
H7B	0.9836	0.3112	0.4719	0.076*	
C8	0.9573 (2)	0.1929 (3)	0.2876 (5)	0.0744 (9)	
H8A	0.9180	0.1876	0.1952	0.089*	
H8B	0.9508	0.1252	0.3589	0.089*	
C9	1.0000	0.4747 (3)	0.2500	0.0527 (10)	
O1	1.0000	0.5804 (2)	0.2500	0.0718 (9)	
S1	0.74489 (6)	0.27612 (8)	0.35060 (12)	0.0737 (4)	

### Atomic displacement parameters (Å^2^)

**Table:**

	*U*^11^	*U*^22^	*U*^33^	*U*^12^	*U*^13^	*U*^23^
C1	0.087 (3)	0.077 (3)	0.083 (2)	−0.033 (2)	0.035 (2)	−0.0259 (19)
C2	0.064 (2)	0.088 (3)	0.093 (3)	−0.0080 (18)	0.0067 (18)	−0.036 (2)
C3	0.0537 (18)	0.0508 (16)	0.0664 (18)	−0.0055 (12)	−0.0001 (14)	−0.0088 (13)
C4	0.0653 (19)	0.0458 (15)	0.0511 (15)	−0.0061 (13)	0.0077 (13)	−0.0088 (13)
C5	0.0605 (18)	0.0406 (14)	0.0497 (15)	−0.0045 (12)	0.0015 (12)	0.0009 (12)
C6	0.0611 (18)	0.0449 (15)	0.0447 (14)	0.0029 (12)	−0.0001 (12)	−0.0012 (11)
C7	0.076 (2)	0.0562 (18)	0.0583 (17)	0.0009 (15)	−0.0001 (15)	0.0126 (14)
C8	0.095 (3)	0.0469 (16)	0.081 (2)	−0.0040 (16)	0.0034 (19)	0.0095 (15)
C9	0.060 (2)	0.047 (2)	0.051 (2)	0.000	−0.0125 (18)	0.000
O1	0.0603 (18)	0.0437 (16)	0.111 (3)	0.000	−0.0167 (16)	0.000
S1	0.0880 (7)	0.0573 (6)	0.0757 (6)	−0.0154 (4)	0.0209 (4)	0.0017 (4)

### Geometric parameters (Å, º)

**Table:**

C1—C2	1.333 (5)	C6—C9	1.484 (3)
C1—S1	1.689 (4)	C6—C7	1.504 (4)
C1—H1	0.9300	C7—C8	1.511 (4)
C2—C3	1.400 (5)	C7—H7A	0.9700
C2—H2	0.9300	C7—H7B	0.9700
C3—C4	1.384 (4)	C8—C8^i^	1.517 (7)
C3—H3	0.9300	C8—H8A	0.9700
C4—C5	1.450 (4)	C8—H8B	0.9700
C4—S1	1.724 (3)	C9—O1	1.227 (5)
C5—C6	1.332 (4)	C9—C6^i^	1.484 (3)
C5—H5	0.9300		
			
C2—C1—S1	112.4 (3)	C9—C6—C7	116.1 (2)
C2—C1—H1	123.8	C6—C7—C8	115.5 (3)
S1—C1—H1	123.8	C6—C7—H7A	108.4
C1—C2—C3	113.5 (3)	C8—C7—H7A	108.4
C1—C2—H2	123.3	C6—C7—H7B	108.4
C3—C2—H2	123.3	C8—C7—H7B	108.4
C4—C3—C2	112.3 (3)	H7A—C7—H7B	107.5
C4—C3—H3	123.9	C7—C8—C8^i^	113.4 (2)
C2—C3—H3	123.9	C7—C8—H8A	108.9
C3—C4—C5	124.9 (3)	C8^i^—C8—H8A	108.9
C3—C4—S1	109.7 (2)	C7—C8—H8B	108.9
C5—C4—S1	125.4 (2)	C8^i^—C8—H8B	108.9
C6—C5—C4	131.8 (3)	H8A—C8—H8B	107.7
C6—C5—H5	114.1	O1—C9—C6	121.53 (16)
C4—C5—H5	114.1	O1—C9—C6^i^	121.53 (16)
C5—C6—C9	117.8 (2)	C6—C9—C6^i^	116.9 (3)
C5—C6—C7	126.1 (3)	C1—S1—C4	92.15 (18)
			
S1—C1—C2—C3	0.1 (4)	C9—C6—C7—C8	−86.3 (3)
C1—C2—C3—C4	−0.2 (4)	C6—C7—C8—C8^i^	73.2 (4)
C2—C3—C4—C5	179.5 (3)	C5—C6—C9—O1	36.8 (3)
C2—C3—C4—S1	0.2 (3)	C7—C6—C9—O1	−143.08 (18)
C3—C4—C5—C6	178.9 (3)	C5—C6—C9—C6^i^	−143.2 (3)
S1—C4—C5—C6	−2.0 (4)	C7—C6—C9—C6^i^	36.92 (18)
C4—C5—C6—C9	178.3 (2)	C2—C1—S1—C4	0.0 (3)
C4—C5—C6—C7	−1.8 (5)	C3—C4—S1—C1	−0.1 (2)
C5—C6—C7—C8	93.8 (4)	C5—C4—S1—C1	−179.4 (2)

Symmetry code: (i) −*x*+2, *y*, −*z*+1/2.

### Hydrogen-bond geometry (Å, º)

**Table:**

*D*—H···*A*	*D*—H	H···*A*	*D*···*A*	*D*—H···*A*
C7—H7*A*···S1	0.97	2.53	3.205 (3)	126
C7—H7*B*···O1^ii^	0.97	2.53	3.282 (3)	135

Symmetry code: (ii) −*x*+2, −*y*+1, −*z*+1.
